# Identification of a pleiotropic locus on chromosome 7q for a composite left ventricular wall thickness factor and body mass index: the HyperGEN Study

**DOI:** 10.1186/1471-2350-10-40

**Published:** 2009-05-09

**Authors:** Weihong Tang, Richard B Devereux, Na Li, Albert Oberman, Dalane W Kitzman, Dabeeru C Rao, Paul N Hopkins, Steven A Claas, Donna K Arnett

**Affiliations:** 1 Division of Epidemiology and Community Health, School of Public Health, University of Minnesota, Minneapolis, MN, USA; 2 Division of Cardiology, Weill Cornell Medical College, New York, NY, USA; 3 Division of Biostatistics, University of Minnesota, Minneapolis, MN, USA; 4 Division of Preventive Medicine, University of Alabama-Birmingham, Birmingham, Alabama, USA; 5 Wake Forest University School of Medicine, Winston-Salem, NC, USA; 6 Division of Biostatistics, Washington University School of Medicine, St Louis, MO, USA; 7 Cardiovascular Genetics, University of Utah, Salt Lake City, UT, USA; 8 Department of Epidemiology, University of Alabama-Birmingham, Birmingham, Alabama, USA

## Abstract

**Background:**

Left ventricular (LV) mass and wall thickness are closely associated with measures of body size and blood pressure and also correlated with systolic and diastolic function, suggesting a contribution of common physiologic mechanisms, including pleiotropic genes, to their covariation.

**Methods:**

Doppler echocardiography was performed in 434 African-American (1344 individuals) and 284 white families (1119 individuals). We conducted a genome-wide linkage scan for LV mass, LV structure and function, and composite factors derived from a factor analysis of LV structure and function in the HyperGEN Study population.

**Results:**

Factor analysis identified (i) a LV wall thickness factor correlated strongly with interventricular septal thickness (IVSTd) and posterior wall thickness (PWTd) and (ii) a LV diastolic filling factor strongly correlated with early and atrial phase peak transmitral filling velocities. The LV phenotypes and composite factor scores were analyzed in multipoint variance components linkage model implemented in SOLAR with 387 microsatellite markers. In whites, the two highest LODs were 3.42 for LV atrial phase peak filling velocity at 144 cM on chromosome 1 and 3.12 for the LV wall thickness factor at 160 cM on chromosome 7. The peak LODs of the component traits (IVSTd and PWTd) clustered at the same region as the composite factor. Adjusting the factor score for body mass index (BMI) substantially reduced the peak LOD at this region (LOD = 1.92). Bivariate linkage analysis of the composite factor with BMI improved LOD to 3.42 at 158 cM. Also in whites, suggestive linkage was observed on chromosomes 2 and 4 for LV mass, chromosomes 3, 5, 10, and 17 for LV atrial phase peak filling velocity, and chromosome 10 for LV diastolic filling factor. In African Americans, suggestive linkage was observed on chromosome 12 for LV mass, chromosome 21 for IVSTd, and chromosome 3 for LV internal diameter at end-diastole.

**Conclusion:**

Our study suggests that a region on chromosome 7 contains pleiotropic genes contributing to the variations of both LV wall thickness and BMI in whites.

## Background

Left ventricular hypertrophy (LVH), due to thickening of LV walls and/or LV chamber enlargement, is a strong predictor of cardiovascular mortality and morbidity [1]. Increase of LV mass is considered to be a compensatory process to maintain systolic LV wall stress in response to physiological and pathological stimuli such as body growth, obesity, and hypertension [2]. Therefore, LV mass and wall thickness are closely associated with measures of body size and blood pressure [3,4]. In addition, LV mass, geometric pattern, and systolic and diastolic function are inter-correlated [5-7], suggesting common physiological links and/or shared genetic influences.

Genetic influences have been demonstrated for measures of LV mass, wall thickness, and function [8-10]. In the Hypertension Genetic Epidemiology Network (HyperGEN) study, bivariate genetic analyses of LV diastolic function with LV structure and systolic function detected shared genetic/familial influences between LV transmitral early (MVE) and atrial phase peak filling velocities (MVA) in both African-American and white hypertensive sibships [8]. In another study, a bivariate genetic analysis of pubertal twins reported common genetic factors for LV mass and weight, explaining more than 90% of correlation between the two traits [9].

Factor analysis is a multivariate data reduction technique that derives latent phenotypes by identifying variation common to a set of correlated variables. It has been shown to be a useful tool in genetic studies to localize genomic regions contributing to clustering of correlated traits, such as metabolic syndrome-related risk variables [11]. In the present study, we conducted a multipoint variance-components linkage analysis of LV mass, LV structure and function, and composite factors derived from factor analysis of LV structure and function, based on data from families participating in the National Heart, Lung, and Blood Institute (NHLBI) HyperGEN Study. Furthermore, we applied bivariate linkage analysis to investigate pleiotropic loci contributing to the correlation between a composite LV wall thickness factor and body mass index (BMI), and between MVA and heart rate.

## Methods

### Population

HyperGEN is one of four Family Blood Pressure Program networks supported by the NHLBI to identify genetic contributions to hypertension [12]. Hypertensive sibships were drawn from population-based cohorts or from the community-at-large. Eligible hypertensive siblings had onset of hypertension by age 60 years and at least one hypertensive sibling who was willing to participate. Hypertension was defined as average systolic blood pressure (SBP) ≥ 140 mm Hg or average diastolic blood pressure (DBP) ≥ 90 mm Hg on at least two different evaluations, or treatment for hypertension. In its second phase, HyperGEN recruited offspring of the hypertensive siblings. The Genetics of Left Ventricular Hypertrophy Study, a study ancillary to HyperGEN, performed echocardiography in four HyperGEN centers (Minneapolis, MN; Salt Lake City, Utah; Forsyth County, NC; and Birmingham, AL). The Institutional Review Board of participating institutions approved the protocol and all participants gave informed consent. Included in the study are 1344 African Americans (913 hypertensive siblings and 431 offspring) and 1119 whites (589 hypertensive siblings and 530 offspring) who had complete data on echocardiography phenotypes and genetic markers.

### Echocardiography

Two-dimensionally (2D) guided M-mode, 2D and Doppler echocardiograms were performed following a standardized protocol developed at the Echocardiography Reading Center at the New York Hospital-Cornell Medical Center [13]. Measurements were made at the central echocardiography core lab as previously described [8]. LV wall thickness and dimension at end-diastole, including LV internal diameter (LVIDd), posterior wall thickness (PWTd), and interventricular septal thickness (IVSTd), were measured according to American Society of Echocardiography recommendations [14,15]. Relative wall thickness (RWT) was calculated by 2*PWTd/LVIDd. LV mass was calculated by an anatomically validated formula that yields values closely correlated with necropsy LV weight (r = 0.90) [16].

LV systolic function was assessed by stress-corrected midwall fractional shortening (MWS). LV diastolic filling parameters were measured by pulsed Doppler in apical four chamber view. MVE and MVA obtained at the mitral valve leaflet tips were used in the analysis.

The reading center has reported good reproducibility of LV measurements by a protocol identical to that used in the HyperGEN (e.g. intraclass correlation coefficients = 0.85 for IVSTd, 0.87 for LVIDd, 0.83 for PWTd, 0.71 for MWS, 0.58 for MVE, and 0.57 for MVA) [17].

### Genotyping

A total of 387 polymorphic markers (Cooperative Human Linkage Center Screening set 8) spaced an average of 9.7 cM apart were genotyped by the Marshfield Clinic's Center for Medical Genetics-Mammalian Genotyping Service in Marshfield, WI. A high-throughput scanning fluorescence detector system was used to genotype short tandem repeat polymorphisms. The average heterozygosity of the markers was 0.76. Marker consistency with Mendelian expectations within families was tested using ASPEX [18], and only confirmed relatives with marker compatibility were used in the linkage analysis.

### Statistical Analysis

Comparison of distributions of demographic and clinical characteristics between race groups was made by generalized estimating equations, which account for familial relatedness. Prior to linkage and factor analyses, each LV variable was adjusted for age, age^2^, and gender in race- and generation-specific linear regression models. Residuals from the linear models were standardized to zero mean and unit variance in each of the four race-by-generation groups. The standardized residuals were included in linkage and factor analyses stratified by race. Participants were excluded from factor analyses if their standardized residuals were ≥ 5 standard deviations (SD) from the means. For linkage analysis, a more stringent exclusion (> 4 SD) was used since linkage analysis is more sensitive to the influence of outliers.

A maximum likelihood-based factor analysis was applied to the residuals of PWTd, IVSTd, LVIDd, stress-corrected MWS, MVA, MVE, and isovolumic relaxation time (IVRT). Details of factor analysis modeling can be found in a supplement (see Additional file 1). In the initial factor analysis of the seven variables, two factors were retained by the eigenvalue ≥ 1.0 criteria and factor loading pattern was consistent between African Americans and whites (see Table S1 of Additional file 1). The first factor had major factor loadings (> 0.4) with PWTd, IVSTd, and stress-corrected MWS and the second factor with MVA and MVE. LVIDd and IVRT correlated weakly with both factors (factor loading < 0.4). To increase the relative contribution of the retained factors and reduce noise from variables with small loadings on the factors, LVIDd and IVRT were excluded from the final factor analysis models. Finally, factor scores, corresponding to the estimated values of the underlying factors, were calculated for each individual based on the final factor pattern and the values of observed phenotypes. To reduce kurtosis, participants with factor scores more than 4 SD from the mean were removed. The final factor scores were approximately normally distributed (both skewness and kurtosis < 0.6) and included in linkage analysis.

Race-stratified multipoint linkage analysis was performed using the variance components approach implemented in Sequential Oligogenic Linkage Analysis Routines (SOLAR) [19]. Details of linkage analysis can be found in the supplement (see Additional file 1). Prior to the linkage analysis, multipoint IBD statistics were computed separately for African Americans and whites by GENEHUNTER version 2.1 [20] and used in the SOLAR linkage analysis. The linkage analysis was conducted for both the individual LV phenotypes and the composite factor scores.

In the linkage analysis, LV phenotypes and factor scores were first analyzed without covariate adjustments. Since these variables have been adjusted for age, age^2^, and gender prior to the factor analysis, the linkage model without covariate adjustment is referred to as minimally adjusted model. Secondly, the following covariates were adjusted for in linkage analysis and this model is referred to as maximally adjusted model: BMI, SBP, diabetes, and current smoking. Heart rate was included in the maximally adjusted model for MVA, MVE, and the factor related to LV diastolic filling parameters. In addition, when a LOD score from the maximally adjusted model was substantially reduced compared to the minimally adjusted model at a position where the minimally adjusted LOD score was above 3.0, each of the covariates was adjusted for separately to delineate the influence of individual covariate adjustment on the linkage signal.

In the above analysis, if adjustment for an individual covariate had important influence on the LOD score for a LV phenotype, it is possible that this indicates pleiotropic genetic effects between the phenotype and covariate. To test this hypothesis, bivariate linkage analyses [21] were conducted between the corresponding phenotype and individual covariate (i.e., BMI and heart rate, as discussed below) on chromosomes where strong linkage signals (i.e., LOD > 3) were obtained in minimally adjusted models and substantial reduction in LOD scores was observed after the covariate adjustment. In addition, bivariate linkage analyses were also conducted among component phenotypes on chromosomes where the corresponding composite factor showed strong linkage (i.e., LOD > 3). In SOLAR, an in-house algorithm was implemented to automatically convert the two degrees of freedom (df) bivariate LOD score to a one df equivalent LOD score. Details of the bivariate linkage model can be found in the supplement (see Additional file 1).

Finally, the SOLAR "lodadj" procedure was used to estimate empirical single-point p-values for the observed univariate and bivariate LODs. Details on this procedure can be found in the supplement (see Additional file 1).

## Results and discussion

Table 1 presents descriptive statistics for clinical and echocardiographic measures by race. All of the risk factors listed in Table 1 differed significantly between African Americans and whites. Family structures are shown in Table 2. On average, white families were larger than African-American families, yielding more relative pairs than African-American families.

**Table 1:** Characteristics of Study Sample: Means ± SD or Percentage (%)

	African American (n = 1344)	White (n = 1119)	P*
Age, years	44.9 ± 13.5	49.2 ± 13.9	< 0.0001
Females, %	65.5	50.6	< 0.0001
Weight, kg	89.9 ± 22.4	84.5 ± 18.3	< 0.0001
Height, cm	167.6 ± 9.0	170.0 ± 9.3	0.039
Body mass index, kg/m^2^	32.0 ± 7.5	29.2 ± 5.9	< 0.0001
Systolic blood pressure, mm Hg	128.9 ± 22.0	122.9 ± 18.7	< 0.0001
Diastolic blood pressure, mm Hg	74.0 ± 11.6	70.8 ± 9.9	< 0.0001
# Hypertensive medication	0.95 ± 0.99	0.75 ± 0.89	< 0.0001
Ejection fraction, %	61.4 ± 7.9	61.9 ± 7.4	0.0147
Interventricular septal thickness, cm	0.92 ± 0.14	0.88 ± 0.13	< 0.0001
Posterior wall thickness, cm	0.86 ± 0.13	0.81 ± 0.13	< 0.0001
Relative wall thickness	0.33 ± 0.06	0.32 ± 0.05	< 0.0001
Left ventricular mass index, g/m^2.7^	41.7 ± 12.1	37.4 ± 10.7	< 0.0001
LV transmitral atrial phase peak filling velocity, cm/sec	72.4 ± 20.2	65.8 ± 18.4	< 0.0001
LV transmitral early peak filling velocity, cm/sec	81.5 ± 18.5	72.8 ± 17.6	< 0.0001
Stress-corrected midwall shortening, %	106.6 ± 12.9	109.6 ± 13.1	< 0.0001
Hypertension, %	71.6	55.9	< 0.0001
Diabetes, %	16.4	9.8	< 0.0001
Current smoking, %	27.4	10.1	< 0.0001

*for age and sex, test was adjusted for familial clustering; for other variables, test was adjusted for age, sex and familial clustering.

**Table 2:** Distribution of Family Structures

	African American	White
Number of families	434	284
Family size: average (range)	3.1 (2–10)	3.9 (2–12)
Number of individuals	1344	1119
Sib pairs	685	937
Half-sib pairs	180	17
Parent-child pairs	363	415
Avuncular pairs	412	618
Half avuncular	92	14
1st cousins	107	237
Half 1st cousins	34	0
Grandparent-grandchild	8	2
Grand avuncular	6	0
Half grand avuncular	2	0

Total relative pairs	1889	2240

Table 3 presents factor-loading patterns from the final factor analysis models. In both race groups, two factors were retained with the eigenvalue ≥ 1.0 criterion. A likelihood ratio test showed that two factors were sufficient to explain the covariance among the five variables (p = 0.5 in whites and 0.8 in African Americans for testing the null hypothesis that two factors are sufficient). The first factor explained 66% of common variance and 41% of total variance in African Americans, and 66% and 39% in whites, respectively. The percentage of common and total variances explained by the second factor was 33% and 20%, respectively, in African Americans, and 34% and 20%, respectively, in whites. Since the first factor correlated strongly with PWTd and IVSTd and the second factor with MVA and MVE, the first factor is labeled a "LV wall thickness factor" and the second labeled a "LV diastolic filling factor."

**Table 3:** Factor Loading Pattern in the Final Factor Analysis Models

	African American		White	
Variables	Factor 1	Factor 2	Factor 1	Factor 2

LV posterior wall thickness	0.95	0.01	0.93	0.03
Interventricular septal thickness	0.91	0.03	0.89	0.05
Stress-corrected midwall shortening	-0.57	0.11	-0.53	0.07
LV transmitral atrial phase peak filling velocity	0.06	0.50	0.08	0.46
LV transmitral early peak filling velocity	-0.07	0.88	-0.10	0.89

Common variance	2.07	1.04	1.97	1.02
% common variance	66.6	33.4	65.9	34.1
% total variance	41.4	20.8	39.4	20.4

Factor 1, named LV wall thickness factor;

Factor 2, named LV diastolic filling factor.

Estimates of heritability ± SE for the LV phenotypes are presented in Table 4.

**Table 4:** Estimates of Heritability ± SE for LV Structure and Function Phenotypes in African American and White Families

Phenotype	African American		White	
	
	Min Adj*	Max Adj**	Min Adj*	Max Adj**
Interventricular septal thickness	0.33 ± 0.07	0.29 ± 0.07	0.42 ± 0.07	0.42 ± 0.07
LV internal diameter	0.51 ± 0.07	0.46 ± 0.07	0.41 ± 0.07	0.37 ± 0.07
Posterior wall thickness	0.33 ± 0.07	0.29 ± 0.07	0.41 ± 0.07	0.40 ± 0.07
Relative wall thickness	0.25 ± 0.07	0.23 ± 0.07	0.54 ± 0.07	0.56 ± 0.07
LV Mass	0.57 ± 0.07	0.58 ± 0.07	0.46 ± 0.07	0.35 ± 0.07
LV transmitral atrial phase peak filling velocity†	0.40 ± 0.07	0.43 ± 0.07	0.40 ± 0.07	0.33 ± 0.07
LV transmitral early peak filling velocity†	0.44 ± 0.07	0.44 ± 0.07	0.43 ± 0.07	0.43 ± 0.07
Stress-corrected midwall shortening	0.26 ± 0.07	0.26 ± 0.07	0.13 ± 0.07	0.11 ± 0.06
LV wall thickness factor	0.36 ± 0.07	0.33 ± 0.07	0.44 ± 0.07	0.44 ± 0.07
LV diastolic filling factor†	0.49 ± 0.07	0.49 ± 0.07	0.45 ± 0.07	0.44 ± 0.07

Estimates of heritability for all traits are significant at p < 0.05;

*Adjusted for age, age^2^, and gender;

**Additionally adjusted for BMI, SBP, diabetes, current smoking, and heart rate (for variables labeled with † only).

Plots for whole genome linkage analysis of the LV structure and function including LV mass are presented in Figures S1 and S2 of the supplements (see Additional files 2, 3 and 4). Table 5 presents phenotypes and chromosomal regions that showed peak LOD > 2.0 from at least one adjustment model. In whites, LV wall thickness factor and MVA showed strongest linkage signals. For the LV wall thickness factor, we observed a highest LOD score of 3.12 (empirical p-value = 0.0002) at 160 cM on chromosome 7 between markers D7S2195 (155 cM) and GATA189C06 (163 cM) in whites. The 1-LOD-unit support interval extends from 154 cM to 167 cM. Adjustment for BMI, SBP, diabetes, and current smoking attenuated the LOD score to 2.24. Among the four covariates, adjustment for BMI was responsible for the reduction in LOD score at this location (LOD = 1.92 after additional adjustment for BMI); adjustment for covariates other than BMI did not result in material change in the LOD (LOD: 3.05 – 3.39). For MVA, a maximum LOD of 3.42 (empirical p-value = 0.00005) was obtained at 144 cM on chromosome 1. Adjustment for the five covariates reduced the LOD score to 2.32. The reduction in LOD was mainly due to the adjustment for heart rate: LOD = 2.50 after additional adjustment for heart rate; adjustment for other covariates than heart rate did not materially change the LOD score (LOD: 3.20 – 3.45). Also in whites, the LV diastolic filling factor showed a suggestive linkage at 28 cM on chromosome 10 (LOD = 2.05) (Table 5). The component phenotypes MVE and MVA both peaked at the same location, with a peak LOD of 1.11 for MVA and 1.60 for MVE. Adjustment for the five covariates did not result in appreciable changes in the LOD scores (data not shown).

**Table 5:** Peak Multipoint LOD Scores and Flanking Markers for Regions with LOD > 2.0 from at Least One Adjustment Model in African Americans and Whites

Trait	Model	Chr	Pos (cM*)	LOD/p†	Flanking Markers (cM*)
African Americans					
IVSTd	Min	21	37	1.32/0.0066	ATA27F01 (37)
IVSTd	Max1	21	38	2.17/0.0005	ATA27F01 (37)/GATA188F04 (40)
LVIDd	Min	3	36	2.30/0.0006	AFM036YB8 (37)
LVIDd	Max1	3	36	2.16/0.0012	AFM036YB8 (37)
LVM	Min	12	18	1.18/0.0109	GATA49D12 (18)
LVM	Max1	12	19	2.13/0.0011	GATA49D12 (18)/GATA11H08 (26)
LVM	Max3	12	18	1.54/0.0051	GATA49D12 (18)
Whites					
LVM	Min	2	170	2.40/0.0004	ATA27H09 (165)/GATA71D01 (173)
LVM	Max1	2	169	1.96/0.0010	ATA27H09 (165)/GATA71D01 (173)
LVM	Max3	2	170	1.94/0.0011	ATA27H09 (165)/GATA71D01 (173)
LVM	Min	4	43	2.34/0.0004	ATA27C07 (43)
LVM	Max1	4	43	1.79/0.0019	ATA27C07 (43)
LVM	Max3	4	43	1.86/0.0016	ATA27C07 (43)
PWTd	Min	7	161	2.51/0.0003	D7S2195 (155)/GATA189C06 (163)
PWTd	Max1	7	162	1.59/0.0028	D7S2195 (155)/GATA189C06 (163)
LV wall thickness factor	Min	7	160	3.12/0.0002	D7S2195 (155)/GATA189C06 (163)
LV wall thickness factor	Max1	7	161	2.24/0.0008	D7S2195 (155)/GATA189C06 (163)
MVA	Min	1	144	3.42/0.00005	ATA29D04 (137)/GATA12A07 (152)
MVA	Max2	1	141	2.32/0.0006	ATA29D04 (137)/GATA12A07 (152)
MVA	Min	3	26	2.28/0.0005	GATA164B08 (26)
MVA	Max2	3	27	2.94/0.0002	GATA164B08 (26)
MVA	Min	5	148	2.56/0.0003	ATA23A10 (148)
MVA	Max2	5	148	2.05/0.0012	ATA23A10 (148)
MVA	Min	10	72	2.17/0.0008	ATA5A04 (63)/AFM329xa9 (76)
MVA	Max2	10	92	2.35/0.0006	GATA121A08 (88)/GATA87G01 (94)
MVA	Min	17	54	2.13/0.0008	GGAA9D03 (51)/GGAA7D11 (57)
MVA	Max2	17	55	2.27/0.0007	GGAA9D03 (51)/GGAA7D11 (57)
LV diastolic filling factor	Min	10	28	2.05/0.0017	ATA31G11 (28)
LV diastolic filling factor	Max2	10	28	1.85/0.0024	ATA31G11 (28)

IVSTd = interventricular septal thickness at end-diastole, LVIDd = LV internal diameter at end-diastole, LVM = LV mass, MVA = LV transmitral atrial phase peak filling velocity, PWTd = LV diastolic posterior wall thickness; *in Kosambi distance; †empirical p value based on simulation;

Min: adjusted for age, age^2^, and gender;

Max1: additionally adjusted for BMI, SBP, diabetes, and current smoking;

Max2: additionally adjusted for heart rate, BMI, SBP, diabetes, and current smoking;

Max3: additionally adjusted for BMI.

**Figure 1:**
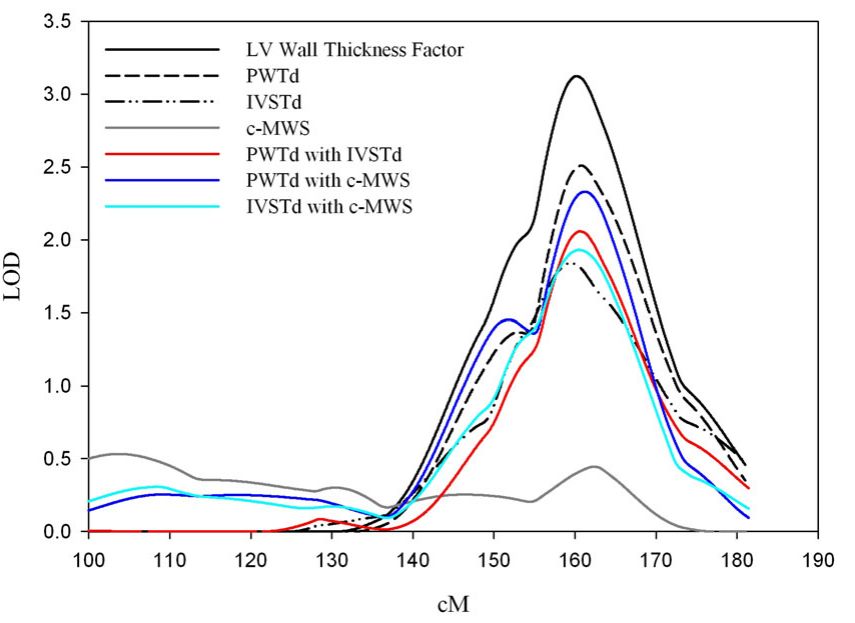
**Multipoint linkage plots for LV wall thickness factor and univariate and bivariate linkage plots for its major component variables on chromosome 7 in whites**. Logarithm of odds (LOD) scores (y-axis) and their respective cM (x-axis) from the p-telomere (left) to the q-telomere (right) are shown. PWTd = posterior wall thickness, IVSTd = interventricular septal thickness, c-MWS = stress-corrected midwall shortening.

Multipoint linkage results for the LV wall thickness factor were compared to LOD plots from univariate and bivariate linkage analyses of the three major component phenotypes at their peak LOD locations on chromosome 7 (Figure 1). The univariate peak LODs of the three phenotypes clustered around the same region as the composite factor: 2.51 for PWTd at 161 cM, 1.83 for IVSTd at 159 cM, and 0.44 for stress-corrected MWS at 162 cM. The peak LODs for PWTd and IVSTd were significant at p < 0.05 (corresponding to LOD > 0.59 without regard to multiple tests). Consistent with the univariate linkage results, the bivariate LOD plots also clustered at this region: peak LOD was 2.06 for PWTd-IVSTd at 160 cM, 2.33 for PWTd-MWS at 161 cM, and 1.93 for IVSTd-MWS at 160 cM (Figure 1).

**Figure 2:**
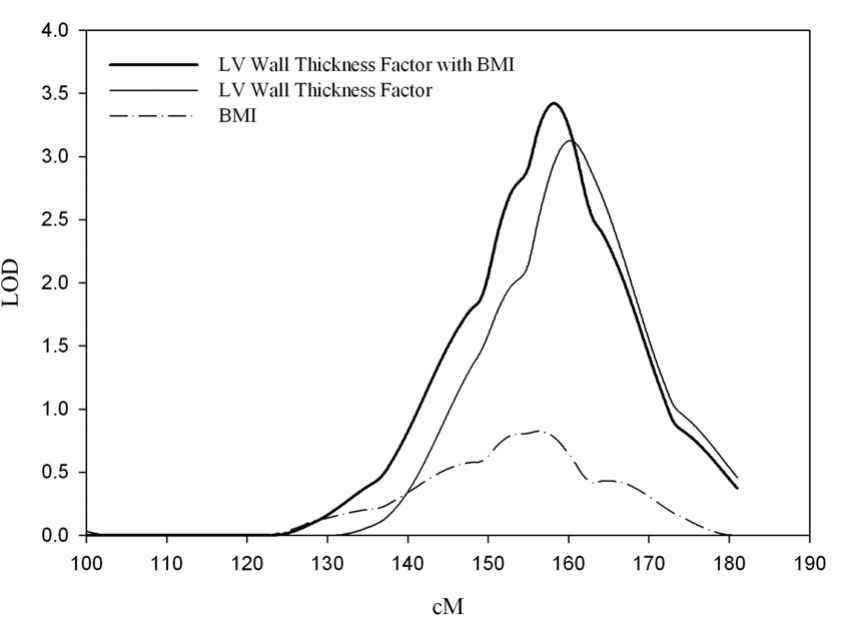
**Bivariate and univariate linkage plots for LV wall thickness factor and body mass index (BMI)**. Same labels for x- and y- axes as in Figure 1.

Bivariate genetic analysis between LV wall thickness factor and BMI found significant genetic (0.28 ± 0.11, p < 0.05) and environmental (0.26 ± 0.09, p < 0.05) correlations between the two phenotypes. Based on these correlations, the proportion of total additive genetic variance and environmental variance that is due to shared genes and shared environment was estimated as 7.8% (i.e., 0.28^2^) and 6.8% (i.e., 0.26^2^), respectively. LOD plots from bivariate and univariate linkage analyses on chromosome 7 are shown in Figure 2. The peak LOD score from bivariate linkage analysis of LV wall thickness factor with BMI was 3.42 (empirical p = 0.00008) at 158 cM, being stronger than the univariate LOD scores of the two individual traits (LOD = 0.82 for BMI at 156 cM). For the QTL at this location, the hypothesis of *ρ*_*Q *_= 0 was rejected (p = 0.0004) and that of *ρ*_*Q *_= 1 could not be rejected (p = 1.0). In the bivariate linkage analysis, further adjustment for SBP, smoking, and diabetes slightly increased the peak LOD to 3.57 (empirical p = 0.00008) at the same location.

There are also significant genetic (0.39 ± 0.11, p < 0.05) and environmental (0.21 ± 0.08, p < 0.05) correlations between MVA and heart rate after adjusting for age, age^2^, and gender. However, bivariate linkage analysis of MVA with heart rate on chromosome 1 yielded a lower LOD (peak LOD = 2.38 at 145 cM) than the single-trait LOD for MVA. Heart rate alone did not show linkage signal at this region (LOD = 0), suggesting that the strong linkage signal observed for MVA in the minimal adjustment model was likely confounded by loading condition measured by heart rate.

To our knowledge, our study is the first to apply a factor analysis approach to investigate the multivariate correlation architecture underlying LV wall thickness, dimension, and systolic and diastolic function. Furthermore, we conducted a multipoint genome-wide scan for the composite factor scores derived from the factor analysis models to localize genetic loci contributing to the clustering of these traits. We observed measures for LV wall thickness (PWTd and IVSTd) strongly loaded on a factor that was also associated with LV systolic function measured by stress-corrected MWS; LV diastolic filling parameters loaded on a different factor. In the linkage analysis, we identified regions on chromosomes 7q36 and 1p13 that showed strong linkage for the LV wall thickness factor and MVA, respectively. The linkage evidence for LV wall thickness factor is further strengthened by the fact that both individual measures of wall thickness strongly mapped to the same region. This locus had pleiotropic effects on body size as measured by BMI. Based on the genome-wide significance criteria (p = 0.000049 and LOD ≥ 3.3) proposed by Lander and Kruglyak [22], the LOD for MVA in minimally adjusted model reached genome-wide significance after correcting for multiple testing. The bivariate LODs for LV wall thickness factor with BMI in both minimally and maximally adjusted models reached borderline significance at the genome-wide level. The strong linkage signal observed for MVA on chromosome 1 was likely confounded by loading condition, as suggested by the results of multivariate adjustment and bivariate linkage analyses with heart rate. It is important to note that the strong linkage evidence obtained in this study needs to be replicated in other populations to validate the linkage findings and rule out false positives. Future gene discovery efforts may not be justified before proper replication is obtained.

At the region on chromosome 7 where the LV wall thickness factor showed strong linkage in whites, LV mass peaked at approximately the same location but with weaker signal in the same population: peak LOD was 0.44 at 156 cM and 0.14 at 155 cM in the minimally and maximally adjusted models, respectively. The difference in magnitude of linkage signal between LV mass and the LV wall thickness factor is likely due to the fact that the locus at this location is mainly for LV wall thicknesses, which is captured at a larger extent by the wall thickness factor than LV mass. Mathematically, the formulae for LV mass [16] includes both wall thickness (IVSTd and PWTd) and dimension (LVIDd) while the LV wall thickness factor is mainly determined by wall thickness. With similar measures of wall thickness, the value of LV mass can differ substantially depending on LV dimension or the extent of LV remodeling (i.e., LV wall thickness relative to dimension). Accordingly, the correlation between LV mass and the LV wall thickness factor is lower (0.66 in whites and 0.71 in African Americans after adjustment for age and gender) compared to the correlation between the LV wall thickness factor and individual measures of LV wall thickness (correlation > 0.90 in both race groups) (see Table S3 of Additional file 1).

In addition to the strong linkage signals for the LV composite factor scores, we also observed suggestive linkage for LV mass and other structure phenotypes on other chromosomes (Table 5). Mayosi et al conducted a genome-wide linkage analysis of electrocardiographic (ECG) and echocardiographic LV phenotypes in 224 white British families (868 individuals) ascertained through a hypertensive proband [23]. In that study, suggestive evidence for linkage was obtained for ECG LV mass on chromosomes 7 (LOD = 1.67 at 120 cM) and 12 (LOD = 2.19 at 78 cM), and for echocardiographic LV mass on chromosome 5 (LOD = 1.6 at 40 cM) [23]. At the three locations, the strongest linkage signal we observed for LV mass in whites of our study was on chromosome 12: peak LOD was 0.93 at 99 cM and 1.28 at 95 cM in the minimally and maximally adjusted models, respectively.

Our observation that LV diastolic filling parameters mainly loaded on a different factor than LV wall thickness and systolic function may reflect differences in the underlying physiologic mechanisms regulating LV diastolic and systolic function. Specifically, the primary physiologic determinant of systolic function is myocardial contractility, whereas it is myocardial relaxation and effective chamber compliance that influence LV diastolic early and late filling, respectively [24-26]. At the cellular level, myocardial contraction and relaxation are related to different phases of cytoplasmic calcium cycling, with the former dependent on release of calcium from sarcoplasmic reticulum (SR) via the calcium release channels and the latter dependent on reuptake of calcium into the SR by the SR-calcium-ATPase pump which is modulated by phospholamban [25,26]. While preload (approximated by heart rate and stroke volume) and afterload (approximated by SBP, DBP, and stroke volume) conditions might be the driving force for the formation of the separate LV diastolic filling factor, this factor had weak correlations with heart rate (whites: -0.06; AAs: -0.09), SBP (whites: 0.06; AAs: 0.07), DBP (whites: -0.05; AAs: -0.06), and stroke volume (whites: 0.13, AAs: 0.18), suggesting that preload and afterload conditions are unlikely the underlying mechanism responsible for the clustering of the LV diastolic filling parameters.

Since body size is an important correlate of LV mass, it is possible that there are pleiotropic genes contributing to these two phenotypes. In our study, the genetic correlation between LV mass and BMI was 0.35 ± 0.08 (p < 0.05) in African Americans and 0.57 ± 0.09 (p < 0.05) in whites after adjustment for age, age^2^, and gender. These data agree with evidence from other genetic studies of LV mass with body size in twins and families [9,27]. For example, Verhaaren et al conducted a bivariate genetic analysis in 341 twins of 11 years old and detected a substantial influence of common genetic factors for the clustering of LV mass and weight, explaining more than 90% of correlation between these traits [9]. In a study of 149 nuclear families comprising healthy adults and children, genetic and/or familial factors accounted for all of the correlation between LV mass and weight in adults and 59% of the correlation in children [27]. Our study further extends these findings by providing evidence for a genetic locus on chromosome 7 that had pleiotropic effects on LV wall thickness and body size. It is important to note that this region has previously been linked to stature and obesity measures in other studies. For height, the peak LOD score was 3.40 at marker D7S2195 (155 cM) in a Swedish population [28] and 2.91 at D7S1826 (162 cM) in a Finnish population [29]. In the HERITAGE Family Study, marker D7S3070 (163 cM) and a marker within the NOS3 gene (164 cM) were reportedly linked to change in abdominal total fat in response to exercise with LODs of 2.53 and 2.34, respectively [30]. In our study, the univariate LOD score for height and bivariate LOD score for LV wall thickness factor-height were 0.35 (163 cM) and 2.27 (160 cM), respectively. Since BMI and height were correlated (r = -0.08, p < 0.05), we cannot rule out the possibility that this locus is also a pleiotropic locus for height.

There are 161 annotated genes in the 1-LOD-unit support interval (154 cM to 167 cM) for the peak LOD region on chromosome 7. Within this interval, we identified two possible candidates which may contribute to the variation of LV wall thickness factor and its correlation with BMI: nitric oxide (NO) synthase 3 (NOS3 or ENOS, 163–164 cM) and AMP-activated non-catalytic protein kinase γ2 (PRKAG2 or AMPK-GAMMA-2, 165–166 cM). NOS3 synthesizes NO and is constitutively expressed in the cardiac myocytes [31]. NO is involved in physiologic processes contributing to LVH, including vasodilation and production of cardiac fibroblast extracellular matrix [32]. As mentioned above, a marker within the NOS3 gene was linked to change in abdominal total fat after exercise in the HERITAGE Family Study [30]. The PRKAG2 gene encodes for the γ2 subunit of AMP-activated protein kinase (AMPK), an enzyme involved in the modulation of glucose uptake and glycolysis [33]. It has been confirmed that mutations in PRKAG2 cause a novel glycogen storage disease of the heart in which hypertrophy, ventricular pre-excitation, and conduction system defects coexist [34]. If the linkage findings on chromosome 7 are replicated in other populations, future studies that use tagging SNP and/or extensive resequencing approaches might be able to delineate the role of these genes in the variation of LV wall thickness and body size and covariation between them.

We also searched the Framingham 100K genome-wide association study [35] database at the NCBI dbGAP website [36] and noticed a SNP rs1177946 in a novel gene CNTNAP2 (156–160 cM on chromosome 7) that showed associations with averaged LV diastolic wall thickness: p = 0.007 after adjustment for age and gender and 0.01 after additional adjustment for other risk factors, including height and weight. This SNP was associated with average BMI at p = 0.03 in the same study. The CNTNAP2 gene encodes a protein named contactin-associated protein-like 2 (CASPR2). CASPR is a member of the neurexin superfamily that mediates cell-cell interactions in the nervous system and is also expressed in the heart. The relevance of CNTNAP2 to LV hypertrophy is unknown. It is important to note that the association of rs1177946 with LV diastolic wall thickness or BMI did not reach statistical significance after adjusting for multiple testing. Therefore, data presented here can be at best considered as hypothesis-generating.

In general, we observed different loci for the LV phenotypes between African Americans and whites. The overall discrepant results may be explained by differences in population characteristics. Specifically, as shown in Table 1, all demographic and clinical characteristics are different between the two groups. The difference in the risk factor profiles may in part reflect difference in genetic etiology and/or environmental modifiers that act to modify the influence of genetic factors. On chromosome 7, there was no signal for the LV wall thickness factor in African Americans. This discrepancy may be explained by anthropometric difference between African Americans and whites, as reflected by larger weight and BMI in African Americans (Table 1). Given that the locus on chromosome 7 is a pleiotropic locus for BMI, we may speculate that the genetic factor related to this locus is more prevalent in African Americans than whites, leading to the manifestation of linkage signals in the former group. In addition, the majority of the participants in our study were hypertensive. As such, the findings of the study might not be able to be replicated in a general population. Moreover, both genetic and environmental correlations between LV mass and BMI and between LV wall thickness factor and BMI were modest, suggesting that shared genetic and environmental factors contribute less to the additive genetic and environmental variations than genetic and environmental factors unique to each phenotype. Finally, factor analysis is a data reduction technique used to summarize correlated variables. The interpretation of factors and factor loading pattern are contingent upon the nature of the phenotypes that are included in the analysis. The derived factor scores are not clinical phenotypes, but instead statistical indexes to indicate where an individual stands on the retained factors. Therefore, factor analysis in genetic studies should be guided by and interpreted in the context of physiology and pathophysiology of the phenotypes.

## Conclusion

We have observed a locus on chromosome 7 that showed strong linkage signals for the clustering of LV wall thicknesses. This locus also had pleiotropic effects on BMI and height. There are plausible candidate genes in this region that may contribute to the increase of LV wall thickness and body size. If the linkage findings are replicated in other populations, fine-scale genotyping of the identified region, including the candidate genes, is needed to identify the specific genes and variants that are responsible for the observed linkage. Identification of the responsible genes offers promise in understanding novel mechanisms and developing therapeutic strategies in the prevention and treatment of LVH.

## Abbreviations

AA: African American; BMI: body mass index; cM: centimorgan; DBP: diastolic blood pressure; df: degrees of freedom; IVRT: isovolumic relaxation time; IVSTd: interventricular septal thickness at diastole; LOD: logarithm of the odds; LV: left ventricular or left ventricle; LVH: left ventricular hypertrophy; LVIDd: left ventricle internal diameter at diastole; MVA: left ventricular transmitral atrial phase peak filling velocity; MVE: left ventricular transmitral early peak filling velocity; MWS: stress-corrected midwall fractional shortening; PWTd: posterior wall thickness at diastole; RWT: relative wall thickness; SBP: systolic blood pressure; SD: standard deviation; SR: sarcoplasmic reticulum.

## Competing interests

The authors declare that they have no competing interests.

## Authors' contributions

WT conceived and designed the study, performed the statistical analyses, and drafted the manuscript. RBD conceived and designed the study and produced the echocardiographic data. NL interpreted the results. AO conceived and designed the study. DCR interpreted the results. SAC interpreted the results and participated in the revision of the manuscript. DWK, PNH, and DKA conceived and designed the study and interpreted the results.

## Pre-publication history

The pre-publication history for this paper can be accessed here:

http://www.biomedcentral.com/1471-2350/10/40/prepub

## Supplementary Material

Additional file 1 **Supplemental text and tables**. This file provides additional statistical methods, results, and legends for supplemental figures. Click here for file

Additional file 2 **Supplemental figure 1**. This file provides multipoint linkage plots for LV mass and LV structure and function phenotypes in whites. Click here for file

Additional file 3 **Supplemental figure 2**. This file provides multipoint linkage plots for LV mass and LV structure and function phenotypes in African Americans. Click here for file

Additional file 4 **Supplemental figures legend**. This file provides legend for Supplemental figures 1 and 2. Click here for file
