# Supplementary material for: Identification of a pleiotropic locus on chromosome 7q for a composite left ventricular wall thickness factor and body mass index: the HyperGEN Study
Source: BMC Med Genet. 2009 May 9;10:40. doi: 10.1186/1471-2350-10-40 (PMC2692848; doi:10.1186/1471-2350-10-40)
Supplement: Additional file 1 — Supplemental text and tables. This file provides additional statistical methods, results, and legends for supplemental figures. [file 1471-2350-10-40-S1.pdf]

Identification of a Pleiotropic Locus on Chromosome 7q for a Composite Left Ventricular Wall Thickness Factor and Body Mass Index: the HyperGEN Study

Weihong Tang, Richard B. Devereux, Na Li, Albert Oberman, Dalane W. Kitzman, Dabeeru C. Rao, Paul N. Hopkins, Steven A. Claas, Donna K. Arnett

Supplement Material

Statistical Analysis

Factor analysis: A maximum likelihood-based factor analysis was applied to the residuals of PWTd, IVSTd, LVIDd, stress-corrected MWS, MVA, MVE, and isovolumic relaxation time (IVRT). In factor analysis model [1], each individual phenotype is expressed as a linear function of a set of latent factors and an error term. The regression coefficient of a phenotype on a latent factor, named factor loading, reflects the relationship of the latent factor to that phenotype. A factor loading of 0.40 with a phenotype, corresponding to 15% of variance explained by the factor, was commonly used to judge meaningful correlation between the factor and the phenotype [1]. Eigenvalues, defined by the sums of the squared factor loadings, are used to evaluate the contribution of each factor to the set of variables (i.e., eigenvalues $\geq$1.0) [1].

Single-trait linkage analysis: Race-stratified multipoint linkage analysis was performed using the variance components approach implemented in Sequential Oligogenic Linkage Analysis Routines (SOLAR) [2]. Significance of a genetic contribution of the QTL (ie, genetic linkage) was tested by comparing the maximum likelihood of a full model to that of a reduced model that constrained the QTL component to be zero. The difference between the two*$\log_{10}$ likelihoods produced a LOD score. Prior to the linkage analysis, multipoint IBD statistics were computed separately for African Americans and whites by GENEHUNTER version 2.1 [3]. The IBD files were then converted into SOLAR format by an in-house program in SOLAR and used in the linkage analysis. Race-specific allele frequencies for the genetic markers were estimated based on the marker allele frequencies of randomly selected, unrelated subjects (232 African Americans and 214 whites) recruited from the same populations as hypertensive siblings.

Bivariate linkage analysis: Bivariate linkage analysis decompose the phenotypic correlation (ρ_P) between two traits that were measured in pairs of relatives into QTL-specific additive genetic (ρ_Q), residual additive genetic (ρ'_g), and environmental (ρ_e) correlations: $\rho_P = \rho_Q\sqrt{h^2_{1Q}h^2_{2Q}} + \rho'_g\sqrt{h'^2_1\,h'^2_2} + \rho_e\sqrt{(1-h^2_1)(1-h^2_2)}$, where h^2_{1Q} and h^2_{2Q} are QTL-specific heritabilities for traits 1 and 2, respectively, and h'^2_1 and h'^2_2 are residual heritabilities for traits 1 and 2, respectively. Significance testing of QTL-specific pleiotropy and complete pleiotropy was made against the null hypothesis: ρ_Q=0 and ρ_Q =1, respectively, by using likelihood ratio tests. Failure to reject the null hypothesis ρ_Q=0 indicates coincident linkage (i.e., no pleiotropy) between two traits and failure to reject the null hypothesis ρ_Q=1 indicates complete pleiotropy at a specific locus.

Simulation in linkage analysis: The SOLAR “lodadj” procedure was used to estimate empirical single-point p-values for the observed univariate and bivariate LODs. Details on this procedure have been described elsewhere [4, 5]. In brief, this procedure simulated Mendelian transmission of a completely-informative marker in the families under the null hypothesis of no linkage with the quantitative traits under investigation. The marker and traits were analyzed to generate the distribution of LODs under the null hypothesis. Based on the null distribution, we computed empirical p-values by the method recommended by North et al. [6]. The number of replication was set to 10,000 for the traits except for MVA, PWTd, LV wall thickness factor, and LV wall thickness factor with BMI. For these 4 traits, 10,000 replications did not yield a LOD higher than the observed peak LODs and therefore more simulations were used to increase the precision (MVA: 20,000; PWTd and LV wall thickness factor: 15,000; LV wall thickness factor with BMI: 25,000).

Results

Table 1. Factor Loading Pattern in the Initial Factor Analysis Models

	African American		White	
Variables	Factor 1	Factor 2	Factor 1	Factor 2
LV posterior wall thickness	0.95	0.08	0.94	0.002
LV interventricular septal thickness	0.91	0.09	0.89	0.03
LV diastolic internal diameter	0.19	0.12	0.08	0.10
Stress-corrected midwall shortening	-0.58	0.08	-0.55	0.11
LV transmitral atrial phase peak filling velocity	0.03	0.48	0.10	0.57
LV transmitral early peak filling velocity	-0.12	0.95	-0.07	0.73
LV isovolumic relaxation time	0.22	-0.18	0.11	-0.25
Common variance	2.16	1.19	2.01	0.94
% common variance	64.4	35.6	68.1	31.9
% total variance	30.9	17.0	28.7	13.4

Factor 1, named LV wall thickness factor;
Factor 2, named LV diastolic filling factor.

Table 2. Comparison of Factor Loading Patterns with and without Varimax Rotation in the Final Factor Analysis Models

	African American		White	
Rotation method/Variables	Factor 1	Factor 2	Factor 1	Factor 2
No rotation				
LV posterior wall thickness	0.95	0.01	0.93	0.03
Interventricular septal thickness	0.91	0.03	0.89	0.05
Stress-corrected midwall shortening	-0.57	0.11	-0.53	0.07
LV transmitral atrial phase peak filling velocity	0.06	0.50	0.08	0.46
LV transmitral early peak filling velocity	-0.07	0.88	-0.10	0.89
Varimax rotation				
LV posterior wall thickness	0.95	0.05	0.93	0.05
Interventricular septal thickness	0.91	0.07	0.89	0.07
Stress-corrected midwall shortening	-0.57	0.09	-0.53	0.06
LV transmitral atrial phase peak filling velocity	0.05	0.50	0.07	0.46
LV transmitral early peak filling velocity	-0.10	0.88	-0.12	0.89

Factor 1, named LV wall thickness factor;
Factor 2, named LV diastolic filling factor.

Table 3. Pearson Correlation Coefficients among LV Wall Thickness Factor, LV Mass, LV Wall Thickness, Dimension, Systolic, and Diastolic Functions in African Americans (above the diagonal) and Whites (below the diagonal) after Adjustment for Covariates*

	LVWTF	LVM	PWTd	IVSTd	LVIDd	c-MWS	MVA	MVE
LVWTF		0.71	0.98	0.94	0.19	-0.58	0.07	-0.07
LVM	0.66		0.72	0.68	0.76	-0.32	0.05	0.05
PWTd	0.97	0.66		0.87	0.21	-0.52	0.07	-0.04†
IVSTd	0.93	0.64	0.83		0.15	-0.49	0.07	-0.03†
LVIDd	0.09	0.71	0.11	0.07		0.02†	0.004†	0.10
c-MWS	-0.55	-0.29	-0.48	-0.45	0.07		0.02†	0.13
MVA	0.08	0.09	0.09	0.10	0.02†	-0.02†		0.45
MVE	-0.10	0.05	-0.05	-0.03†	0.08	0.09	0.41	

*Covariates included age, age^2, and gender;
†Not significantly different from 0 at $p < 0.05$, all others were significant at $p < 0.05$;
LVWTF=LV wall thickness factor, LVM = LV mass, PWTd = LV posterior wall thickness, IVSTd = LV Interventricular septal thickness, LVIDd = LV diastolic internal diameter, c-MWS= Stress-corrected midwall shortening, MVA = LV transmitral atrial phase peak filling velocity, MVE = LV transmitral early peak filling velocity.

Figure Legends (see separate files)

Figure 1. Multipoint linkage plots for LV mass and LV structure and function phenotypes in whites. Logarithm of odds (LOD) scores (y-axis) and their respective cM (x-axis) from the p-telomere (left) to the q-telomere (right) are shown. Linkage for LV mass is shown for every chromosome; for other echocardiographic phenotypes, only linkage curves for chromosomes where peak LOD > 1.5 are shown.

Figure 2. Multipoint linkage plots for LV mass and LV structure and function phenotypes in African Americans. Legends are the same as in Figure 1.

References

1. Hatcher L: *A step-by-step approach to using the SAS system for factor analysis and structural equation modeling*, Cary, NC: SAS Institute Inc.; 1994.
2. Almasy L, Blangero J: **Multipoint quantitative-trait linkage analysis in general pedigrees**. *Am J Hum Genet* 1998, **62**:1198-1211.
3. Pratt SC, Daly MJ, Kruglyak L: **Exact multipoint quantitative-trait linkage analysis in pedigrees by variance components**. *Am J Hum Genet* 2000, **66**:1153-1157.
4. Blangero J, Williams JT, Almasy L: **Variance component methods for detecting complex trait loci**. *Adv Genet* 2001, **42**:151-181.
5. Blangero J, Williams JT, Almasy L: **Robust LOD scores for variance component-based linkage analysis**. *Genet Epidemiol* 2000, **19 Suppl 1**:S8-14.

6. North BV, Curtis D, Sham PC: **A note on the calculation of empirical P values from Monte Carlo procedures**. *Am J Hum Genet* 2002, **71**:439-441.
