# Supplementary figures and images for: Identification of a pleiotropic locus on chromosome 7q for a composite left ventricular wall thickness factor and body mass index: the HyperGEN Study
Source: BMC Med Genet. 2009 May 9;10:40. doi: 10.1186/1471-2350-10-40 (PMC2692848; doi:10.1186/1471-2350-10-40)

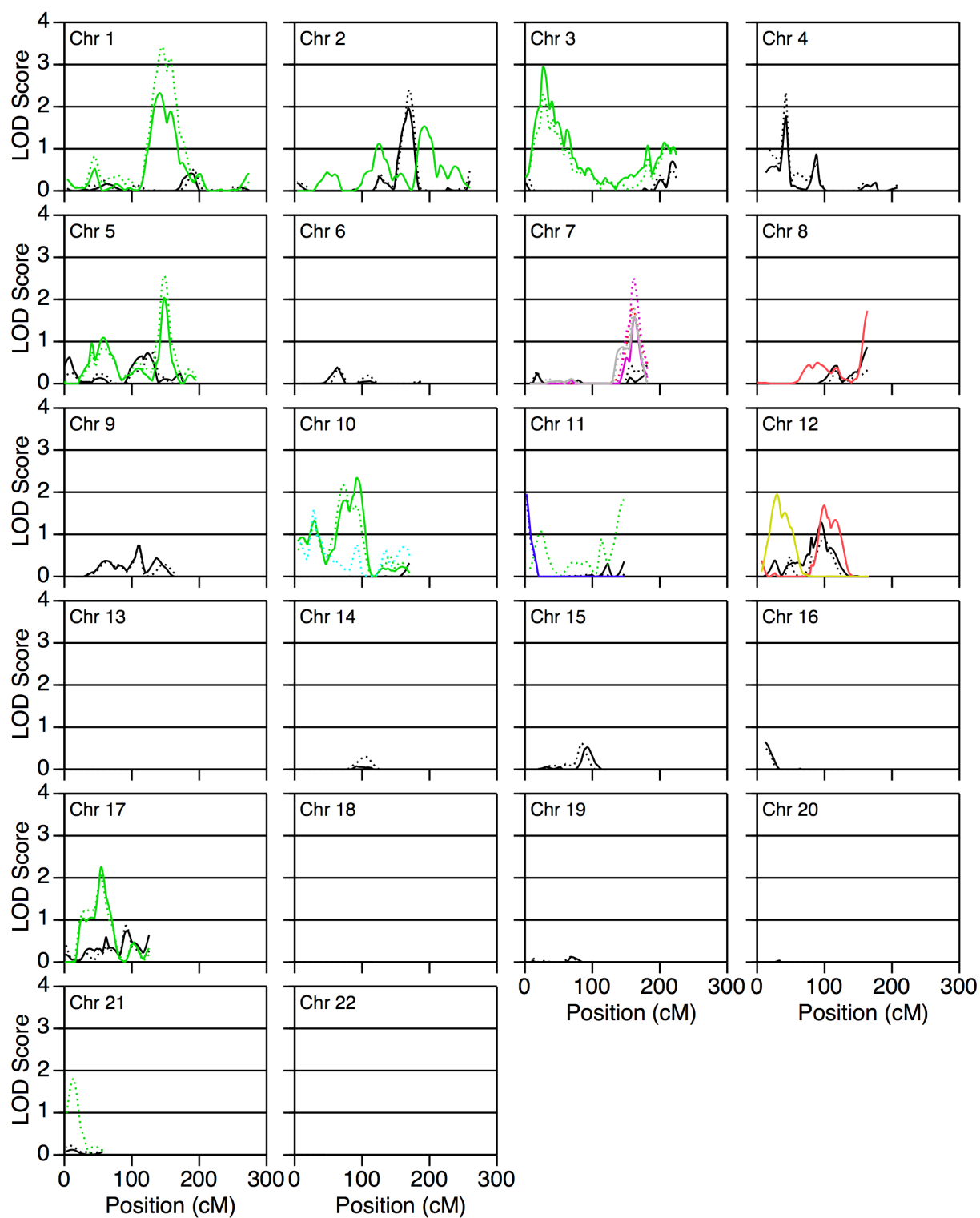

Online Supplements: Figure 1. [Whites]

Supplement: Additional file 2 — Supplemental figure 1. This file provides multipoint linkage plots for LV mass and LV structure and function phenotypes in whites. [file 1471-2350-10-40-S2.pdf]

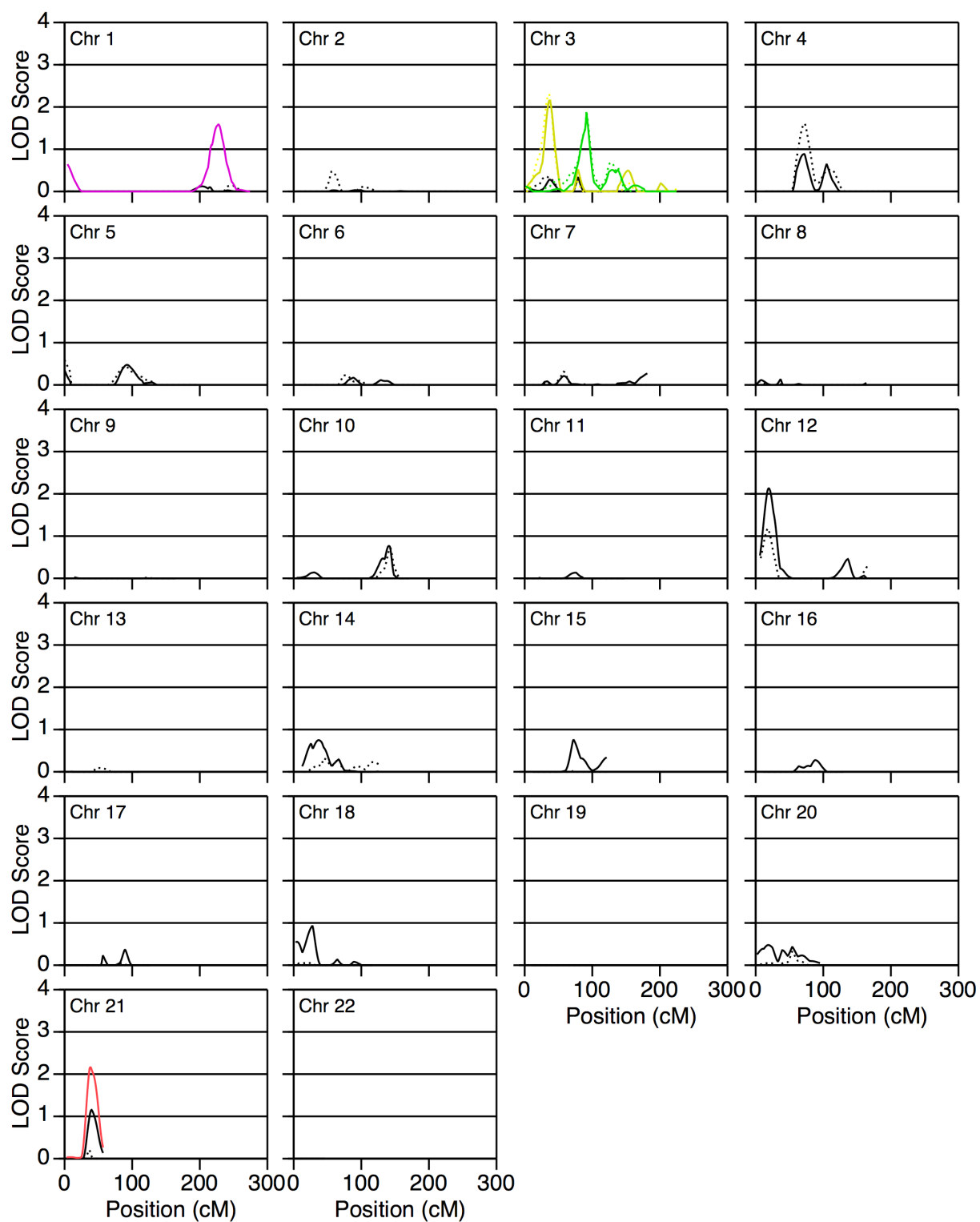

Online Supplements: Figure 2. [African Americans]

Supplement: Additional file 3 — Supplemental figure 2. This file provides multipoint linkage plots for LV mass and LV structure and function phenotypes in African Americans. [file 1471-2350-10-40-S3.pdf]
