# Supplementary material for: Identification of a pleiotropic locus on chromosome 7q for a composite left ventricular wall thickness factor and body mass index: the HyperGEN Study
Source: BMC Med Genet. 2009 May 9;10:40. doi: 10.1186/1471-2350-10-40 (PMC2692848; doi:10.1186/1471-2350-10-40)
Supplement: Additional file 4 — Supplemental figures legend. This file provides legend for Supplemental figures 1 and 2. [file 1471-2350-10-40-S4.pdf]

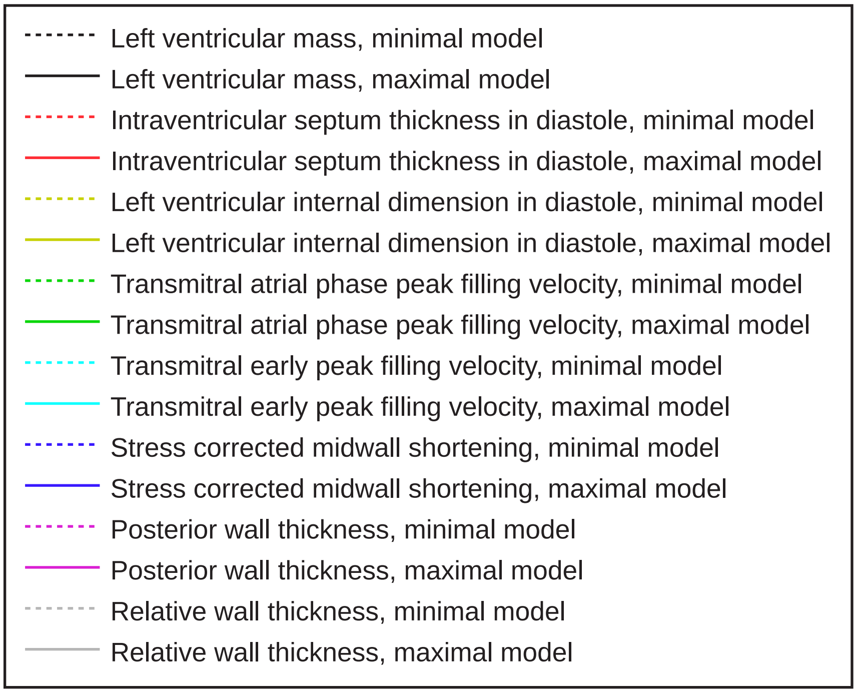
Left ventricular mass, minimal model
Left ventricular mass, maximal model
Intraventricular septum thickness in diastole, minimal model
Intraventricular septum thickness in diastole, maximal model
Left ventricular internal dimension in diastole, minimal model
Left ventricular internal dimension in diastole, maximal model
Transmitral atrial phase peak filling velocity, minimal model
Transmitral atrial phase peak filling velocity, maximal model
Transmitral early peak filling velocity, minimal model
Transmitral early peak filling velocity, maximal model
Stress corrected midwall shortening, minimal model
Stress corrected midwall shortening, maximal model
Posterior wall thickness, minimal model
Posterior wall thickness, maximal model
Relative wall thickness, minimal model
Relative wall thickness, maximal model
